# Remarks upon the Different Modes of Treating the Pedicle in Ovariotomy—Suggestion of a New Method, with Report of an Illustrative Case

**Published:** 1869-06

**Authors:** J. F. Miner


ART. III.—*Remarks upon the different modes of treating the Pedicle in Ovariotomy—Suggestion of a new method, with report of an illustrative case.* BY J. F. MINER, M. D.

The results of the operation of ovariotomy already attained, most fully justify the removal of these tumors by the methods now practiced, so that at the present time there is no propriety in discussing the question of justification in removing by abdominal section, cystic disease of the ovary. A few years since, would-be conservative men, opposed the operation altogether, claiming that palliative measures so often removed or postponed the more serious symptoms that the extra hazard of an operation for radical cure was not to be accepted, but recent improved methods of procedure, united with more extensive observation, have placed ovariotomy with the other established operations of surgery. The results are now so favorable that it may not merely be proposed, but should be urged as offering equal chances with most other capital operations. The questions now remaining to be answered are, what are the best modes of procedure? What plan of operation will insure the greatest attainable success? All the steps of the operation are sufficiently settled, except the one of how to treat the pedicle, and this is really the most important part of the undertaking.

Successes have attended all the various methods of treating the pedicle, so that at present no one of the plans can be wholly thrown aside, but doubtless much of success depends upon the selection. Nothing has yet been proposed, which is not open to objection, and a general desire is manifest in the professional mind for selecting something better.

Ligating with silk or silver wire and returning to the abdomen, must leave the products of inflammation, and to some extent, of decomposition within the abdominal cavity; if the wire or silk is cut short, these substances also remain to be disposed of, and furnish additional sources of danger, while if they are left out through the angle of the wound they prevent union—may admit air to the abdominal cavity and are in some other respects objectionable. If the pedicle is short, this plan may be resorted to, and though not satisfactory, is still often attended with success, and may be the best that can be done. If the pedicle is long and small, it may be left in the lower angle of the wound, and retained with the pins used to close the wound, thus allowing the ligature to separate and the strangulated stump to suppurate outside the cavity of the abdomen; this is a most excellent method of operation, and in cases suited to its adoption, would be chosen above all others by a great majority of operators.

The more recent mode of division with the actual cautery has gained considerable favor, both in Europe and this country, and flattering results have been obtained. Statistics, however, do not thus far show that it has any superiority over former plans, and if statistics should show greater proportion of recoveries they might be deceptive, since it is only applicable to cases where the vessels of the pedicle are small and easily closed, and must necessarily be confined to the simpler and most favorable cases. Division with the cautery may be, and probably is, a valuable addition to the various modes from which surgeons select in making this operation according to the different cases they have to treat, but there are several important objections to it, such as all surgeons will readily appreciate. In the first place, actual cautery will not control hemorrhage in vessels of any considerable size. Again, vessels thus closed are liable to become unsealed and allow of hemorrhage after the return of pedicle and closure of wound. If these objections are removed, we have yet others similar to those urged when ligature is used and pedicle returned, viz.: the products of inflammation and the seared, crisped tissues, must be removed or in some way provided for; these are liable to provoke inflammation and suppuration and afford material for purulent infection. This method, though open to serious objection, is still attended by favorable results. I have been disappointed in its power to control hemorrhage, and have had cases in which it worked admirably. I have observed both its success and failure in my own hands, and in the hands of my associates. Prof. James P. White of Buffalo, has tried it repeatedly and very successfully, and seems to prefer it in suitable cases to all other methods of treating the pedicle yet proposed. I have had opportunity to test by experience all, or nearly all, the different plans thus far suggested, and have been impressed with a growing conviction that nothing yet tested would be long practiced, that something better would take the place of all the present methods. The great improvements which have been made in the operation within the last few years, and the encouraging results which have attended these improved methods, suggest the possible attainment of yet other and still greater advancements; undoubtedly more settled and better determined plans of procedure will insure more uniformly favorable results.

I have proposed for myself, and desire to suggest to others, a plan of separating the tumor from its attachments to the pedicle which appears to my mind as feasible, at least in some instances, and where practicable, as having decided advantages. A few months since I was invited to remove an immense ovarian tumor occurring in the person of Mrs. Foster of Cattaraugus county, N. Y. It was of years standing, had been repeatedly tapped, but at length the contents proved too thick to be drawn through even the largest size canula, and the distress becoming too great for endurance, any operation which would end it, whatever might be the result, was gladly accepted. The tumor was multilocular, very large, weighing, as near as could be ascertained, between seventy-five and one hundred pounds. It was attached throughout its entire circumference to the omentum, intestines, walls of the abdomen, and all other parts in which it came in contact. These attachments were not so firm but that they could be broken up, and with great care the tumor was separated from the surrounding parts until the pedicle was reached. The process of enucleation had been carried on so extensively and successfully that encouragement was afforded for continued trial; the pedicle was large and extended over a wide surface, but by gentle and patient effort it was separated from its entire attachment to the tumor, and the immense growth removed without the ligation of a single vessel. The terminal branches of the vessels of the pedicle gave out no more blood than issued from the vessels of the attachment elsewhere, and there appeared no more occasion for ligature here than elsewhere. All hemorrhage soon ceased, and the incision was closed by interrupted suture.

The success of this procedure was complete, and the patient continued for more than two weeks without a single untoward symptom, so long that her recovery seemed almost certain. She now commenced to lose her relish for food, grew weak and desponding, and died from exhaustion on the twenty or twenty-first day after the operation. The fatal termination of the operation detracted nothing from the success of this mode of treating the pedicle; indeed, so remarkable was the size and attachment of the tumor that any attempt at recovery is surprising, and yet the feeble, emaciated, exhausted patient, continued to improve long enough to show that the manner of treating the pedicle was at least in her case unobjectionable. Upon these facts is partly based the suggestion that the pedicle in ovariotomy can, in some cases at least, be separated without ligature or cautery, thus avoiding many dangers attending it. At first this proposition will appear startling, and surgeons who have tied large vessels in the operation, or who have witnessed the fearful hemorrhage which sometimes takes place from slipping of clamp or ligature, will regard it with surprise, and perhaps without trial look upon it as wholly impracticable. I should myself, probably, be among this number, had I not had opportunity to demonstrate to my own mind certainly, its entire feasibility. The ovarian tumor is generally composed of a firm, dense, fibrous cyst, containing fluid of very varied color and composition. It may, and it may not, have a solid portion, but usually it does have more or less of a body, the remnant of an enlarged or degenerated gland. Upon the surface of this smooth, dense cyst, is spread out the vascular, fibrous, cellular and other tissues which compose the pedicle, but only the terminal branches of the vessels enter this cyst; the vessels may be quite large at their origin, but soon they are numerously divided and enter the cyst, if at all, of capillary size. The attachment of the pedicle to the cyst is more easily broken than any one would mistrust who has not attempted its separation in the manner described, and I am confident that the same efforts which are made to break up adhesions to the peritoneum, omentum and other parts, if extended to the pedicle, will, many times, be equally successful.

If this method can be adopted without hemorrhage or other difficulty, its advantages are apparent. The pedicle can then be returned into the abdominal cavity without any of the objections which have been urged against this procedure. There is no ligature to be discharged by the ulcerative process, or to become encysted, or to induce inflammation. There are no purulent or inflammatory products to be in any way removed or provided for; the pedicle is wholly a living tissue, and has no irritative qualities which render its return to the abdominal cavity objectionable. This cannot be said of it when treated by any other known method.

It is probable that all cases are not suited to this mode of procedure; indeed, it may be only a small proportion which can be disposed of by this method, but that in some instances this plan can be carried out, is to my mind certain. The inquiry is often made, how do you *prefer* to treat the pedicle in ovariotomy? and a brief answer to this very pertinent question will close my suggestions on the subject.

First, then, I would enucleate the tumor without ligature. If I found this impracticable, I would, secondly, apply ligature and fasten the pedicle in the lower angle of wound with the suture pins, leaving ligature outside the pins, so that the slough and all the products of inflammation should be external. The pedicle being short, and vessels small, I would, thirdly, select without much choice, between the actual cautery and arrest of hemorrhage upon the principle of acu-pressure, that is, a needle with ligature attached, passed transversely through the pedicle, weaving it in such manner as to make pressure upon the vessels sufficient to arrest the circulation. Where this method can be made effectual, it approaches very near in point of favor to enucleation without ligature, since the needle can doubtless be very early removed, and the parts beyond the point of its introduction need not be strangulated or the vitality destroyed so as to produce slough. Ligature applied and pedicle returned with the ligature extending out of the lower angle of wound, or cut short and allowed to take care of itself, or each vessel ligated separately and pedicle returned, and all similar methods are not to be chosen, they may be accepted as a final resort where all other resources fail. I have not spoken of the clamp, which is sometimes applied and allowed to rest outside the wound, retaining the grasp of the pedicle. It is scarcely worth while to speak of it, for no good can come of its being used and thus retained.

My object will be fully answered provided the feasibility of enucleation in ovariotomy is shown with sufficient clearness to obtain trial by surgeons. It is not expected that all cases will admit of it, but it is believed that some will, and that where practicable it is the least objectionable of all known methods. Again, the idea of arresting hemorrhage in the pedicle upon the principle of acu-pressure is worthy of consideration. I have never seen or heard of its being suggested or tried, but from what is known of the efficacy of pressure applied in this manner, in arresting hemorrhage elsewhere, it is reasonable to presume that it can be made available in the operation, and if so, that it may have advantages worth knowing.

